# Erythropoiesis-stimulating agent dosing, haemoglobin and ferritin levels in UK haemodialysis patients 2005–13

**DOI:** 10.1093/ndt/gfw043

**Published:** 2016-03-25

**Authors:** Kate Birnie, Fergus Caskey, Yoav Ben-Shlomo, Jonathan A.C. Sterne, Julie Gilg, Dorothea Nitsch, Charles Tomson

**Affiliations:** 1 School of Social and Community Medicine, University of Bristol, Canynge Hall, 39 Whatley Road, Clifton, Bristol BS8 2PS, UK; 2 UK Renal Registry, Southmead Hospital, Bristol, UK; 3 Renal Unit, Southmead Hospital, North Bristol NHS Trust, Bristol, UK; 4 Department of Non-communicable Disease Epidemiology, London School of Hygiene & Tropical Medicine, London, UK; 5 Department of Renal Medicine, Freeman Hospital, Newcastle upon Tyne, UK

**Keywords:** anaemia management, erythropoiesis-stimulating agents, ESA, haemodialysis, haemoglobin

## Abstract

**Background:** Erythropoiesis-stimulating agents (ESAs) with intravenous iron supplementation are the main treatment for anaemia in patients with chronic kidney disease. Although observational studies suggest better outcomes for patients who achieve higher haemoglobin (Hb) levels, randomized controlled trials comparing higher and lower Hb targets have led to safety concerns over higher targets and to changes in treatment guidelines.

**Methods:** Quarterly data from 2005 to 2013 were obtained on 28 936 haemodialysis patients from the UK Renal Registry. We examined trends in ESA use and average dose, Hb and ferritin values over time and Hb according to the UK Renal Association guideline range.

**Results:** The average ESA dose declined over time, with sharper decreases of epoetin seen towards the end of 2006 and from 2009. Average Hb for patients on ESAs was 114.1 g/L [95% confidence interval (CI) 113.7, 114.6] in the first quarter of 2005, which decreased to 109.6 g/L (95% CI 109.3, 109.9) by the end of 2013. Average serum ferritin was 353 µg/L (95% CI 345, 360) at the start of 2005, increasing to 386 µg/L (95% CI 380, 392) in the final quarter of 2013. The percentage of patients with Hb in the range of 100–120 g/L increased from 46.1 at the start of 2005 to 57.6 at the end of 2013.

**Conclusions:** Anaemia management patterns for haemodialysis patients changed in the UK between 2005 and 2013. These patterns most likely reflect clinician response to emerging trial evidence and practice guidelines. Registries play an important role in continued observation of anaemia management and will monitor further changes as new evidence on optimal care emerges.

## INTRODUCTION

Nearly 57 000 adult patients were receiving renal replacement therapy (RRT) in the UK at the end of 2013 (prevalence 888/million), and demand for RRT is rising [1]. Anaemia is a common complication for patients with chronic kidney disease (CKD), resulting in reduced quality of life, fatigue, decreased exercise capacity and shortness of breath. Erythropoiesis-stimulating agents (ESAs) are the main treatment for CKD patients with anaemia [2] and are effective in correcting and maintaining haemoglobin (Hb) levels and decreasing the need for blood transfusions. Intravenous (IV) iron supplements are an important adjunct in the treatment of anaemia in patients on haemodialysis.

Even partial treatment of anaemia with ESAs is expensive, and full correction of Hb requires much higher doses than partial correction. When ESAs were first introduced into clinical practice, randomized controlled trials (RCTs) were not performed, as it was thought unnecessary and even unethical to leave a control group with untreated anaemia. However, the question of whether full correction of anaemia is preferable to partial correction has now been tested in several RCTs. Besarab *et al.* [3] randomized haemodialysis patients with cardiovascular comorbidity to full or partial correction of anaemia: this trial was terminated early when it became clear that benefit from full correction was highly improbable. The US Food and Drug Administration later classified the trial results as showing increased risk of all-cause mortality for the group randomized to the higher Hb target [4] and a later reanalysis of the full trial report showed no improvement in quality of life [5]. Three large RCTs in patients with CKD not yet on dialysis have been completed [6–8] and found no evidence of benefits of a higher (compared with lower) Hb target on cardiovascular events or a composite outcome of death, myocardial infarction, hospitalization for congestive heart failure and stroke, or they found increased risk of adverse events. These conclusions are difficult to reconcile with data from numerous observational studies that demonstrate the best outcomes are in patients who have high Hb concentrations but require only low doses of ESAs (130–140 g/L) [9–11]. One interpretation is that ESA-sensitive patients derive survival benefit from full correction of anaemia, but that patients with ESA resistance (which is a predictor of poor survival) may be harmed by the high doses of ESAs prescribed under the trial protocols in the (often unsuccessful) attempt to normalize Hb concentration.

The target Hb for patients with CKD in the UK Renal Association Clinical Practice Guidelines (3rd edition, 2002–07) was >100 g/L with no suggested maximum [12]. These were altered to 105–125 g/L (4th edition, 2007–09) [13] and lowered to 100–120 g/L in the latest version (5th edition, 2010) [2] following publication of the RCTs in 2006 [6, 8] and 2009 [7]. The impact of these guideline changes on ESA dosing and hence on achieved Hb levels remains unclear. We, therefore, examined trends in ESA dosing in UK haemodialysis patients between 2005 and 2013 and corresponding trends in achieved Hb levels. We related these trends to changes in the national guidelines for management of anaemia.

## METHODS

We identified RCTs and treatment guidelines with the potential to affect anaemia management for patients on haemodialysis during the period 2005–13. These were used to construct a timeline depicting when changes in management were expected.

The UK Renal Registry (UKRR) collects clinical and biochemical data from all patients ≥18 years of age receiving RRT in the UK. Data are extracted electronically, on a quarterly basis, and are validated and cleaned prior to analysis [14]. Some centres submit data extracts that include ESA doses. All patients who received haemodialysis in 2005–13 in centres submitting ESA doses were included in the analyses. Centres reporting fewer than 60% of haemodialysis patients being treated with ESAs were considered to have incomplete data and their data for that quarter were excluded. Patients from 37 centres across England, Wales and Northern Ireland were included in the analyses (from a total of 62 centres in these regions). A patient was considered to be incident during their first year of haemodialysis.

Patients were defined as being on an ESA if a drug type (darbepoetin, epoetin-α, -β or not otherwise specified, or methoxy polyethylene glycol-epoetin β) and/or a dose was recorded. ESA dose is presented as the weekly epoetin dose in our analysis. Doses of ≤150 IU/week (likely to be darbepoetin) were harmonized with epoetin data by multiplying by 200. No adjustments were made with respect to route or frequency of administration. The measurement of Hb (g/L) that was closest to the end of the quarter was extracted. Serum ferritin was measured in µg/L. Data were also extracted on age, sex, race (White, South Asian, Black, Chinese, other or missing), year of starting RRT and primary renal disease (diabetes, glomerulonephritis, hypertension, polycystic kidneys, pyelonephritis, renal vascular disease, other or uncertain [15]).

### Statistical methods

For each calendar year, we derived descriptive statistics summarizing the demographic and clinical characteristics of the cohort. ESA dose and ferritin were log transformed, as they had positively skewed distributions. The percentage of patients on the different ESA drug types were calculated for each quarter. To examine trends in anaemia management over time, we calculated the geometric mean of the ESA dose [with 95% confidence interval (CI)] per quarter, stratified by drug type [(i) darbepoetin and (ii) epoetin-α, -β or not otherwise specified; there were not enough numbers to also include a methoxy polyethylene glycol-epoetin β group]. The doses were also examined restricting to incident patients. The mean achieved Hb levels were calculated separately for ESA-treated and non-treated patients. We calculated the proportion of all patients on ESA treatment over time and the proportion of patients with Hb in the categories <100, 100–120 and >120 g/L. Patients were also put into categories according to their ESA status: those always receiving ESAs over the time period, patients changing on/off ESAs over the time period and those never receiving ESAs. Trends in these statistics over time were compared with the timeline of events with the potential to affect anaemia management. All means and proportions were standardized by year using direct standardization according to the age, sex, race and primary renal disease distribution for all years of data combined. The numbers with missing values for these factors were reported and people with missing values were excluded from the analyses. All analyses were carried out using Stata version 13 (StataCorp, College Station, TX, USA).

## RESULTS

### Timeline of events that may have affected anaemia management

**FIGURE 1:**
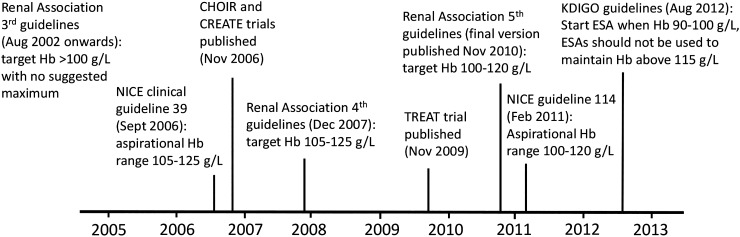
Timeline of events.

Events that may have affected anaemia management in the UK are summarized in Figure 1. The CHOIR trial, published in 2006 [8], randomized 1432 patients with CKD not on RRT to a Hb target of 135 or 113 g/L. There was evidence of increased risk of the primary outcome (a composite of death, myocardial infarction and hospitalization for heart failure or stroke) in the high-Hb compared with the low-Hb group [hazard ratio (HR) 1.34 (95% CI 1.03, 1.74)]. In the CREATE trial, also published in 2006 [6], 603 patients with CKD not on RRT were randomized to a target Hb of 130–150 or 105–115 g/L. Early complete correction of anaemia did not reduce the risk of cardiovascular events. In the TREAT trial, which was the largest RCT and was published in 2009 [7], 4038 patients with diabetes, CKD and anaemia were randomly assigned to achieve a Hb level of ∼130 g/L with the use of darbepoetin alfa or placebo (patients in the placebo group received darbepoetin alfa as a rescue agent if Hb <90 g/L). The risk of a composite outcome of death or cardiovascular event was similar in the two groups [HR for darbepoetin alfa versus placebo 1.05 (95% CI 0.94, 1.17)], but there was a higher incidence of stroke in the darbepoetin alfa arm [HR 1.92 (95% CI 1.38, 2.68)]. Following publication of these RCTs, the target Hb for patients with CKD was lowered to 100–120 g/L in UK Renal Association and National Institute for Health and Care Excellence (NICE) treatment guidelines [2, 16]. The Kidney Disease: Improving Global Outcomes (KDIGO) 2012 guidelines suggest that ESA therapy should be started when Hb is between 90 and 100 g/L for adult dialysis patients and that ESAs should not be used to maintain an Hb concentration >115 g/L in adult patients with CKD, but that individualization of therapy will be necessary as some patients may have improvements in quality of life at an Hb concentration >115 g/L and will be prepared to accept the risks [17].

### Characteristics of the study cohort

A total of 28 936 haemodialysis patients were included in the cohort. A total of 1659 patients (5.9%) never received ESAs within the study period (2005–13), 6648 (23.0%) changed on/off ESAs over the time period and 20 593 (71.2%) always received ESA therapy. Darbepoetin was the drug used most frequently (51.3%), followed by epoetin-β (21.8%), epoetin-α (17.3%), epoetin not otherwise specified (7.4%) and methoxy polyethylene glycol-epoetin β (2.3%). Demographic characteristics of the yearly cohorts are shown in Table 1. The number of patients included in the cohort almost doubled over time, but the age, sex and race distributions changed very little. In 2013, the median age of patients was 66.9 years (interquartile range 54.1–76.8), with 7726 (61.3%) male and 10 095 (80.7%) of White race. The proportion of patients with diabetes as the primary renal disease increased over time, from 17.4% of patients in 2005 to 21.8% in 2013. The number of incident patients included increased steadily over time from *N* = 1423 in 2005 to *N* = 2193 in 2013.

**Table 1:** Characteristics of the cohort by year

	2005	2006	2007	2008	2009	2010	2011	2012	2013
Number of patients									
Incident	1423	1645	1703	1840	1895	1870	1842	1960	2193
Total (including incident)	6930	7896	8637	9419	10 326	10 648	10 824	11 462	12 609
Age, years, median (IQR)	65.5 (51.5–75.0)	66.0 (52.1–75.4)	65.9 (52.4–75.5)	66.1 (52.9–75.7)	66.4 (53.2–75.9)	66.7 (53.7–76.2)	66.8 (53.5–76.5)	67.0 (53.9–76.6)	66.9 (54.1–76.8)
Sex, *n* (%)									
Male	4216 (60.8)	4880 (61.8)	5346 (61.9)	5784 (61.4)	6262 (60.6)	6471 (60.8)	6579 (60.8)	7037 (61.4)	7726 (61.3)
Female	2714 (39.2)	3016 (38.2)	3291 (38.1)	3635 (38.6)	4064 (39.4)	4177 (39.2)	4245 (39.2)	4425 (38.6)	4883 (38.7)
Race, *n* (%)									
White	5477 (83.6)	6286 (84.1)	6884 (83.4)	7580 (83.4)	8419 (83.2)	8783 (83.2)	8822 (81.9)	9489 (82.9)	10 095 (80.7)
Black	295 (4.5)	328 (4.4)	367 (4.5)	400 (4.4)	467 (4.6)	457 (4.3)	514 (4.8)	499 (4.4)	779 (6.2)
South Asian	677 (10.3)	751 (10.1)	882 (10.7)	983 (10.8)	1073 (10.6)	1143 (10.8)	1263 (11.7)	1266 (11.1)	1403 (11.2)
Chinese	39 (0.6)	41 (0.6)	46 (0.6)	45 (0.5)	50 (0.5)	55 (0.5)	51 (0.5)	49 (0.4)	68 (0.5)
Other	64 (1.0)	70 (0.9)	75 (0.9)	81 (0.9)	105 (1.0)	125 (1.2)	128 (1.2)	138 (1.2)	160 (1.3)
Missing	378	420	383	330	212	85	46	21	104
Primary renal disease, *n* (%)									
Diabetes	1182 (17.4)	1400 (18.0)	1581 (18.6)	1792 (19.4)	2004 (19.7)	2065 (19.6)	2191 (20.4)	2351 (20.7)	2718 (21.8)
Glomerulonephritis	1027 (15.1)	1184 (15.2)	1287 (15.2)	1389 (15.0)	1509 (14.8)	1565 (14.9)	1577 (14.7)	1700 (15.0)	1875 (15.1)
Hypertension	386 (5.7)	400 (5.2)	458 (5.4)	510 (5.5)	599 (5.9)	637 (6.1)	642 (6.0)	747 (6.6)	864 (6.9)
Polycystic kidneys	491 (7.2)	565 (7.3)	605 (7.1)	656 (7.1)	704 (6.9)	732 (7.0)	702 (6.5)	772 (6.8)	827 (6.6)
Pyelonephritis	725 (10.6)	814 (10.5)	948 (11.2)	992 (10.7)	1056 (10.4)	1035 (9.8)	1024 (9.6)	1046 (9.2)	1141 (9.2)
Renal vascular disease	561 (8.2)	640 (8.2)	678 (8.0)	702 (7.6)	736 (7.2)	780 (7.4)	769 (7.2)	801 (7.1)	767 (6.2)
Other	965 (14.2)	1113 (14.3)	1184 (13.9)	1345 (14.5)	1485 (14.6)	1598 (15.2)	1667 (15.5)	1776 (15.6)	1955 (15.7)
Uncertain	1477 (21.7)	1652 (21.3)	1751 (20.6)	1865 (20.2)	2079 (20.4)	2115 (20.1)	2156 (20.1)	2167 (19.1)	2314 (18.6)
Missing	116	128	145	168	154	121	96	102	148

### Use of ESAs, 2005–13

**FIGURE 2:**
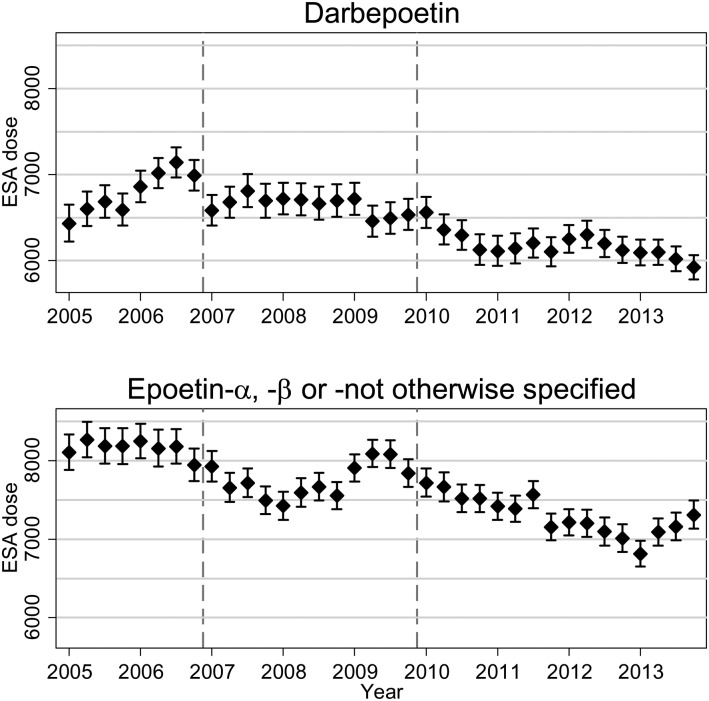
Geometric mean ESA weekly dose and 95% CI. The dashed vertical lines indicate the publication of the CHOIR and CREATE RCTs (2006) and TREAT (2009).

**FIGURE 3:**
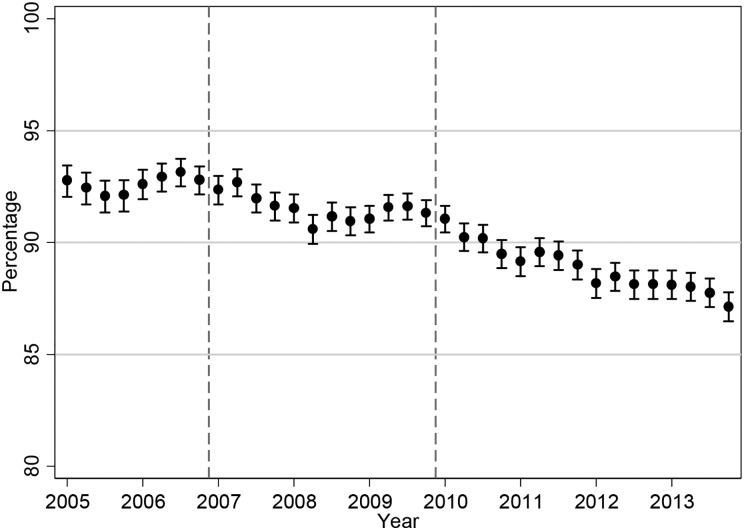
Percentage use of ESAs with 95% CIs, in all haemodialysis patients. The dashed vertical lines indicate the publication of the CHOIR and CREATE RCTs (2006) and TREAT (2009).

Prior to 2007, when existing guidelines had no upper limit to Hb levels, the geometric mean dose of ESA remained stable for epoetin drugs and showed a steady increase for darbepoetin (Figure 2), while the proportion of patients on ESAs remained relatively constant (Figure 3). Near the end of 2006 and in early 2007, there appears to be a decline in the proportion of patients on ESAs, with a modest dose reduction, most likely in response to the joint publication of the NICE guideline [18] and the CHOIR and CREATE trials [4, 6] (see Figure 1). The decline in patients treated with ESAs continues until 2013 [percentage of patients receiving ESAs decreases from 92.8 (95% CI 92.0, 93.5) in the first quarter of 2005 to 87.1 (95% CI 86.5, 87.8) in the final quarter of 2013]. There is an overall decline in average darbepoetin dose from mid-2006. Average doses of epoetin drugs declined from 2006, followed by a rise in 2008–09, followed by a second decline from around 2010 after the publication of the TREAT trial [7] and further published guidelines. The average darbepoetin dose declined from 6431 IU/week (95% CI 6221, 6649) in the first quarter of 2005 to 5921 IU/week (95% CI 5780, 6066) in the last quarter of 2013. The average epoetin dose declined from 8107 IU/week (95% CI 7884, 8336) in the first quarter of 2005 to 7310 IU/week (95% CI 7133, 7491) in the last quarter of 2013. The percentage of patients on the different ESA drug types is shown in the online supplement (Supplementary data, Figure S1). Similar ESA dose patterns were observed in incident patients (Supplementary data, Figure S2).

### Achieved Hb and ferritin, 2005–13

**FIGURE 4:**
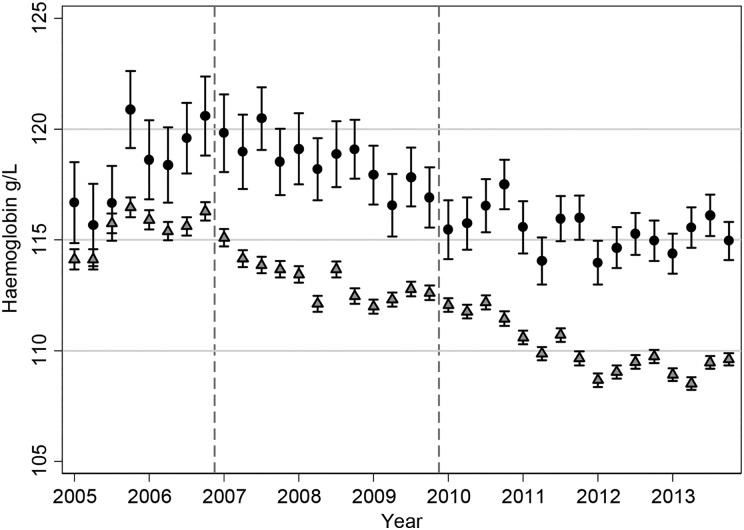
Mean Hb levels over time with 95% CIs in all haemodialysis patients. Circles represent patients not receiving ESAs; triangles represent patients receiving ESAs. The dashed vertical lines indicate the publication of the CHOIR and CREATE RCTs (2006) and TREAT (2009).

**FIGURE 5:**
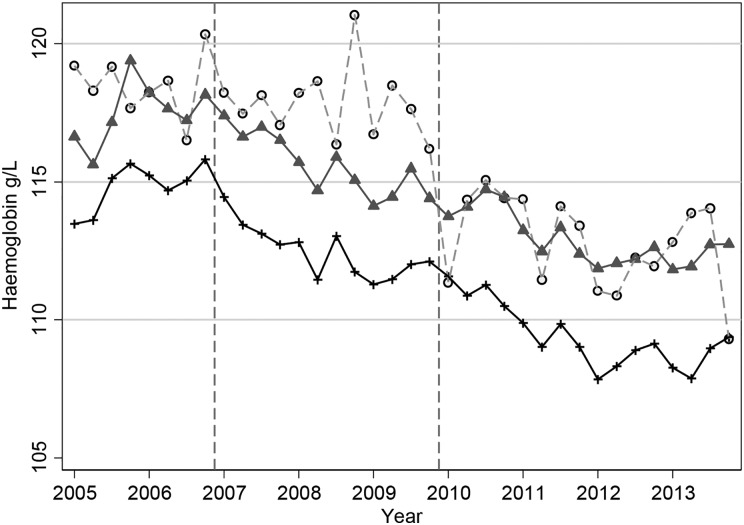
Mean Hb levels over time in all haemodialysis patients by ESA status. Crosses represent patients always receiving ESAs; triangles represent patients changing on/off ESAs over the time period; circles represent patients never receiving ESAs. The dashed vertical lines indicate the publication of the CHOIR and CREATE RCTs (2006) and TREAT (2009).

**FIGURE 6:**
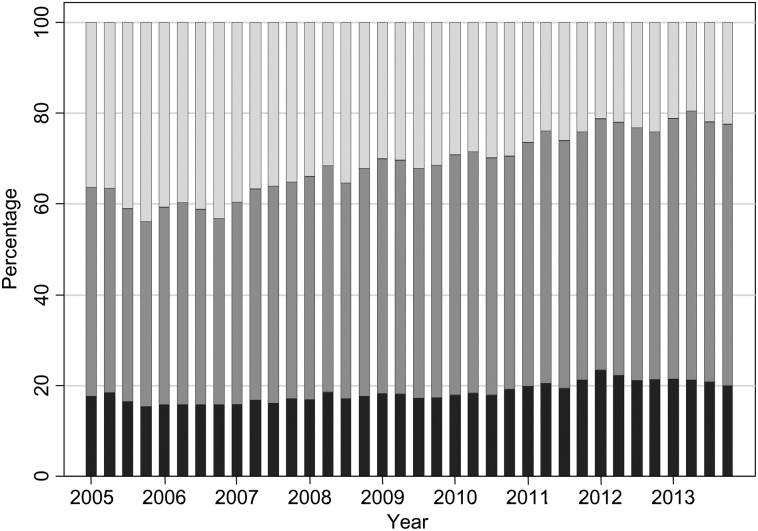
Percentages in the Hb groups (<100, 100–120 and >120 g/L) in all haemodialysis patients. Dark grey represents Hb <100 g/L, mid-grey represents Hb 100–120 g/L and light grey represents Hb >120 g/L.

**FIGURE 7:**
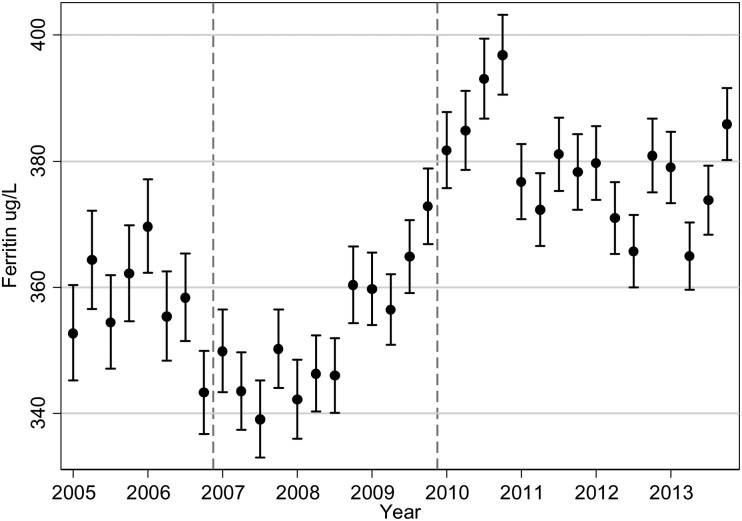
Geometric mean ferritin with 95% CI in all haemodialysis patients. The dashed vertical lines indicate the publication of the CHOIR and CREATE RCTs (2006) and TREAT (2009).

The Hb patterns for patients on ESAs mirror what is seen with ESA dosage, so that we observed an initial small increase in mean Hb until the end of 2006 followed by a steady decline (Figure 4). The mean Hb for patients on ESA in the first quarter of 2005 was 114.1 g/L (95% CI 113.7, 114.6) and was 109.6 g/L (95% CI 109.3, 109.9) by the end of 2013. The decline in mean Hb appears to start in 2007, following NICE guidelines [18] and the publication of the CHOIR [8] and CREATE [6] trials in 2006. The decrease in average Hb levels is then observed for many years—when further guidelines and the TREAT trial [7] were published—until a settling of Hb levels in 2012 and 2013. The lowering in Hb was seen for patients who were receiving ESAs, those not on treatment and across treatment groups (always on ESAs, changing on/off ESAs, never on ESAs; Figure 5), although the numbers of patients never receiving ESAs were small and the pattern showed large fluctuations. The percentage of patients with Hb >120 g/L has decreased over time, from 36.3 at the start of 2005 to 22.4 at the end of 2013 (Figure 6). The percentage of patients with Hb in the range of 100–120 g/L has increased from 46.1 at the start of 2005 to 57.6 at the end of 2013. From the second quarter of 2011 onwards, >55% of haemodialysis patients have had Hb levels in the range of 100–120 g/L. In contrast to average Hb levels, there has been a sharp increase in ferritin levels from 2008 to 2011 (Figure 7), with a decline near the beginning of 2011, after which levels appear to have stabilized. The average serum ferritin was 353 µg/L (95% CI 345, 360) in the first quarter of 2005 and 386 µg/L (95% CI 380, 392) in the final quarter of 2013.

## DISCUSSION

### Summary of findings

We examined ESA doses and Hb levels in haemodialysis patients from 2005 to 2013 in the UK. This period included the publication of major clinical trials and changes to treatment practice guidelines. The use of ESAs has decreased over time, but it is not possible to determine which events had the most influence on dosing, as results from RCTs and anaemia management guidelines were also published during this time. The average Hb level decreased over time, with levels after 2011 largely compliant with the UK Renal Association 2010 target range of 100–120 g/L. A decrease in Hb levels over time was observed for both patients who were treated with ESAs and those not receiving ESAs, suggesting that after the reduction in the Hb target thresholds, patients were started on ESAs at lower levels of Hb. A small, but sharp increase in ferritin levels was seen from 2008 to 2011, coinciding with decreases in ESA use. This pattern cannot be directly explained by adherence to revised anaemia management guidelines. Serum ferritin is a marker of iron balance, i.e. iron loss from occult bleeding, blood sampling and loss of blood in the dialysis machine, versus dietary absorption plus IV supplementation. The observed pattern of ferritin may have been driven by coincident economic pressures to improve the cost-effectiveness of ESA use as well as by emerging evidence that IV iron was safe and effective [19]. Alternatively, it could be that clinicians are administrating the same amount of iron to patients, but it is not being used up so quickly because of reduced ESA doses. Interestingly, the last 2 years have seen a stable pattern with no increase or decrease in levels.

### Comparison with other studies

Results from RCTs and clinical practice patterns in the USA have influenced anaemia management in recent years. A study of US non-dialysis chronic kidney disease patients found that the emergence of safety concerns following RCTs and the subsequent changes in product labelling, restrictions to reimbursement for ESAs and changes to clinical practice guidelines all appeared to influence physician dosing practices, resulting in less frequent use of ESAs, lower ESA doses and lower achieved Hb levels [20]. A study of US haemodialysis patients from the United States Renal Data System found that ESA dosing decreased from 2007 and that Hb levels mirrored ESA dosing trends [21]. The Dialysis Outcomes and Practice Patterns Study (DOPPS) Practice Monitor was developed to detect and report on trends in dialysis care before, during and after implementation of the End-Stage Renal Disease Prospective Payment System, which was initiated by the US Centers for Medicare and Medicaid Services in January 2011. The percentage of patients with Hb >120 g/L declined from 32 in August 2010 to 14 in December 2012. The mean prescribed dose of patients receiving IV epoetin decreased from 20 500 U/week in August 2010 to 13 300 U/week in December 2012, with the greatest decline occurring during June–August 2011. An increase of iron use was observed in 2011 and serum measures of iron stores increased [22]. However, the increase in average IV iron dose did not persist beyond 2011. The sustained increase in ferritin levels in US dialysis patients after policy changes in 2011 appeared to be partly due to reductions in ESA dosing and not solely IV iron dosing practices [23]. In Germany, dialysis procedure rates were changed in 2002 from per-session to weekly flat rate payments, and quality assurance was introduced in 2009 with defined treatment targets for different indices including Hb. DOPPS phases 1–4 (1998–2011) found that Hb levels increased starting in 1998 and remained consistent through 2005, with only 8–10% of patients below 100 g/L. Around 90% of patients were prescribed ESAs, with the dose declining since peaking in 2006. IV iron use was highest in 2011 [24].

### Strengths and weaknesses

The UK Renal Registry is a large and highly representative database allowing trends in clinical practice patterns to be captured [11]. This study gives an indication that clinicians are responding to changes in guidelines important for patient safety. The analysis of ESA usage is, however, limited by incomplete data returns, as some renal centres do not routinely record computerized data on ESA dose or drug type. Centres reporting <60% of haemodialysis patients being treated with ESAs were considered to have incomplete data and were excluded from the analysis. These exclusion criteria are relatively arbitrary, but they are based in part on the frequency distribution graph of the centres' ESA use as it appears in the data. The percentage of patients on ESAs is calculated from these data and incomplete data returns risk impacting on any conclusions drawn. The dose conversion used for darbepoetin may not accurately estimate weekly epoetin dose. There is no information on how much iron therapy was being used to treat anaemia in this population; however, data were available on ferritin levels, giving an indication of achieved iron stores over the period. Data were only obtained from haemodialysis patients and are not generalizable to peritoneal dialysis patients or other CKD populations.

## CONCLUSIONS

Anaemia management patterns for haemodialysis patients have changed in the UK between 2005 and 2013, with these changes likely to have been in response to results from RCTs and changing clinical practice guidelines. There has been a decrease in ESA use, dose and average achieved Hb levels in recent years, with fewer patients having Hb levels >120 g/L and more patients having Hb levels within the 100–120 g/L target range. Average serum ferritin levels have increased in the same time period. Previous research suggests that clinicians are responsive to clinical guidelines, though the degree to which new practices are adopted can vary considerably depending on the context [25, 26]. It is likely that in this case, economic considerations due to the price of ESAs further facilitated adoption of the guidelines, as clinicians could reduce unit expenditure by reducing ESA dosage and treating fewer patients. The continued monitoring of anaemia management through registry data is important for patient safety and enhancing patient quality of life and enables us to ensure that any new evidence on the optimal use of ESA therapy for haemodialysis patients is translated into everyday clinical care.

## Supplementary Material

Supplementary Data

## References

[1] Rao A, Casula A, Castledine C. UK Renal Registry 17th Annual Report: Chapter 2 UK Renal Replacement Therapy Prevalence in 2013: national and centre-specific analyses. Nephron 2015; 129 (Suppl 1): 31–56 25695806 10.1159/000370272

[2] Mikhail A, Shrivastava R, Richardson D. Clinical Practice Guidelines. Anaemia of CKD, 5th edn http://www.renal.org/guidelines (1 July 2015, date last accessed) UK Renal Association, 2010

[3] Besarab A, Bolton WK, Browne JK The effects of normal as compared with low hematocrit values in patients with cardiac disease who are receiving hemodialysis and epoetin. N Engl J Med 1998; 339: 584–590 9718377 10.1056/NEJM199808273390903

[4] FDA Drug Safety Communication: modified dosing recommendations to improve the safe use of Erythropoiesis-Stimulating Agents (ESAs) in chronic kidney disease. http://www.fda.gov/Drugs/DrugSafety/ucm259639.htm2011 (11 August 2015, date last accessed)

[5] Coyne DW The health-related quality of life was not improved by targeting higher hemoglobin in the Normal Hematocrit Trial. Kidney Int 2012; 82: 235–241 22437411 10.1038/ki.2012.76 PMC3388517

[6] Drueke TB, Locatelli F, Clyne N Normalization of hemoglobin level in patients with chronic kidney disease and anemia. N Engl J Med 2006; 355: 2071–2084 17108342 10.1056/NEJMoa062276

[7] Pfeffer MA, Burdmann EA, Chen CY A trial of darbepoetin alfa in type 2 diabetes and chronic kidney disease. N Engl J Med 2009; 361: 2019–2032 19880844 10.1056/NEJMoa0907845

[8] Singh AK, Szczech L, Tang KL Correction of anemia with epoetin alfa in chronic kidney disease. N Engl J Med 2006; 355: 2085–2098 17108343 10.1056/NEJMoa065485

[9] Levin A, Djurdjev O, Duncan J Haemoglobin at time of referral prior to dialysis predicts survival: an association of haemoglobin with long-term outcomes. Nephrol Dial Transplant 2006; 21: 370–377 16249203 10.1093/ndt/gfi209

[10] Regidor DL, Kopple JD, Kovesdy CP Associations between changes in hemoglobin and administered erythropoiesis-stimulating agent and survival in hemodialysis patients. J Am Soc Nephrol 2006; 17: 1181–1191 16565261 10.1681/ASN.2005090997

[11] Macdougall IC, Tomson CR, Steenkamp M Relative risk of death in UK haemodialysis patients in relation to achieved haemoglobin from 1999 to 2005: an observational study using UK Renal Registry data incorporating 30,040 patient-years of follow-up. Nephrol Dial Transplant 2010; 25: 914–919 19934090 10.1093/ndt/gfp550

[12] Standards and Audit Subcommittee of the Renal Association. Treatment of Adults and Children with Renal Failure. Standards and Audit Measures, 3rd edn London: Royal College of Physicians, 2002

[13] Cassidy M, Richardson D, Jones C. Clinical Practice Guidelines. Module 2: Complications, 4th edn http://www.renal.org/guidelines (1 July 2015, date last accessed) UK Renal Association, 2007

[14] Ansell D, Tomson CR. UK Renal Registry 11th Annual Report (December 2008): Chapter 15 The UK Renal Registry, UKRR database, validation and methodology. Nephron Clin Pract 2009; 111 (Suppl 1): c277–c285 19542703 10.1159/000210004

[15] UK Renal Registry 17th Annual Report: Appendices. Nephron 2015; 129 (Suppl 1): 267–322 25695817 10.1159/000370283

[16] National Institute for Health and Care Excellence. Anaemia Management in People with Chronic Kidney Disease. NICE Clinical Guideline 114 http://www.nice.org.uk/guidance/cg114. NICE, 2011.

[17] Kidney Disease: Improving Global Outcomes (KDIGO) Anemia Work Group. KDIGO Clinical Practice Guideline for Anemia in Chronic Kidney Disease. Kidney Int Suppl 2012; 2: 279–335

[18] National Institute for Health and Care Excellence. Anaemia Management in People with Chronic Kidney Disease. NICE Clinical Guideline 39 https://www.nice.org.uk/guidance/cg39. NICE, 2006.

[19] Susantitaphong P, Alqahtani F, Jaber BL Efficacy and safety of intravenous iron therapy for functional iron deficiency anemia in hemodialysis patients: a meta-analysis. Am J Nephrol 2014; 39: 130–141 24513913 10.1159/000358336

[20] Regidor D, McClellan WM, Kewalramani R Changes in erythropoiesis-stimulating agent (ESA) dosing and haemoglobin levels in US non-dialysis chronic kidney disease patients between 2005 and 2009. Nephrol Dial Transplant 2011; 26: 1583–1591 20861195 10.1093/ndt/gfq573

[21] Freburger JK, Ng LJ, Bradbury BD Changing patterns of anemia management in US hemodialysis patients. Am J Med 2012; 125: 906–914.e9 22938926 10.1016/j.amjmed.2012.03.011

[22] Fuller DS, Pisoni RL, Bieber BA The DOPPS practice monitor for U.S. dialysis care: update on trends in anemia management 2 years into the bundle. Am J Kidney Dis 2013; 62: 1213–1216 24140369 10.1053/j.ajkd.2013.09.006

[23] Karaboyas A, Zee J, Morgenstern H Understanding the recent increase in ferritin levels in United States dialysis patients: potential impact of changes in intravenous iron and erythropoiesis-stimulating agent dosing. Clin J Am Soc Nephrol 2015; 10: 1814–1821 26286925 10.2215/CJN.02600315 PMC4594068

[24] Kleophas W, Karaboyas A, Li Y Changes in dialysis treatment modalities during institution of flat rate reimbursement and quality assurance programs. Kidney Int 2013; 84: 578–584 23636176 10.1038/ki.2013.143

[25] Grimshaw JM, Russell IT Effect of clinical guidelines on medical practice: a systematic review of rigorous evaluations. Lancet 1993; 342: 1317–1322 7901634 10.1016/0140-6736(93)92244-n

[26] Grol R, Dalhuijsen J, Thomas S Attributes of clinical guidelines that influence use of guidelines in general practice: observational study. BMJ 1998; 317: 858–861 9748183 10.1136/bmj.317.7162.858 PMC31096

