# Supplementary material for: Erythropoiesis-stimulating agent dosing, haemoglobin and ferritin levels in UK haemodialysis patients 2005–13
Source: Nephrol Dial Transplant. 2016 Mar 25;32(4):692–8. doi: 10.1093/ndt/gfw043 (PMC5410985; doi:10.1093/ndt/gfw043)
Supplement: Supplementary Data [file gfw043_Supplementary_Data.zip › gfw043supp.docx]

**Supplement for: Erythropoiesis stimulating agent dosing, haemoglobin and ferritin levels in UK haemodialysis patients 2005-2013**

**Figure S1 Percentage of patients on the different erythropoiesis stimulating agent (ESA) drugs by quarter**

**Figure S2 Geometric mean ESA weekly dose and 95% confidence interval for incident patients, stratified by drug type**

The dashed vertical lines indicate the publication of the CHOIR and CREATE RCTs (2006) and TREAT (2009)
